# Transcriptome analysis of transgenic apple fruit overexpressing microRNA172 reveals candidate transcription factors regulating apple fruit development at early stages

**DOI:** 10.7717/peerj.12675

**Published:** 2021-12-22

**Authors:** Zhe Zhou, Yanmin Zhu, Hengtao Zhang, Ruiping Zhang, Qiming Gao, Tiyu Ding, Huan Wang, Zhenli Yan, Jia-Long Yao

**Affiliations:** 1 Zhengzhou Fruit Research Institute, Chinese Academy of Agricultural Sciences, Zhengzhou, China; 2 Tree Fruit Research Laboratory, United States Department of Agriculture, Agricultural Research Service, Wenatchee, WA, USA; 3 The New Zealand Institute for Plant & Food Research Limited, Auckland, New Zealand

**Keywords:** Apple, Transgenic fruit, Fruit size, Transcriptome, Transcription factors, Coexpression netword

## Abstract

**Background:**

MicroRNA172 (miR172) has been proven to be critical for fruit growth, since elevated miR172 activity blocks the growth of apple (*Malus x domestica* Borkh.) fruit. However, it is not clear how overexpression of miR172 affects apple fruit developmental processes.

**Methods:**

To answer this question, the present study, analyzed global transcriptional changes in miR172-overexpressing (miR172OX) and nongenetically modified wild-type (WT) apple fruit at two developmental stages and in different fruit tissues via RNA-seq. In addition, two cultivars, ‘Hanfu’ and ‘M9’, which have naturally fruit size variation, were included to identify miR172-dependent DEGs. qRT–PCRwas used to verify the reliability of our RNA-seq data.

**Results:**

Overexpression of miR172 altered the expression levels of many cell proliferation- and cell expansion-related genes. Twenty-four libraries were generated, and 10,338 differentially expressed genes (DEGs) were detected between miR172OX and WT fruit tissues. ‘Hanfu’ and ‘M9’ are two common cultivars that bear fruit of different sizes (250 g and 75 g, respectively). Six libraries were generated, and 3,627 DEGs were detected between ‘Hanfu’ and ‘M9’. After merging the two datasets, 6,888 candidate miR172-specific DEGs were identified. The potential networks associated with fruit size triggered traits were defined among genes belonging to the families of hormone synthesis, signaling pathways, and transcription factors. Our comparative transcriptome analysis provides insights into transcriptome responses to miR172 overexpression in apple fruit and a valuable database for future studies to validate functional genes and elucidate the fruit developmental mechanisms in apple.

## Introduction

Apple (*Malus x domestica*), one of the most widely cultivated fruit trees worldwide, bears fruits of different sizes that may vary several-fold within species (Yao et al., 2015). Fruit size, as a readily apparent and fundamental trait for horticultural crops, is closely associated withcommercial value. Driven by market preferences and economic interests, most apple breeders and growers strive to produce larger-sized fruit. The genetic background of the cultivar is the most critical factor in regulating and determining the final fruit size (Whiting, Ophardt & McFerson, 2006). Genes that are involved in fruit size regulation generally function by controlling cell number and cell size (Harada et al., 2005; Sugimoto-Shirasu & Roberts, 2003).

For cell number regulation, the first two identified and cloned fruit weight QTLs were *FW2.2* and *FW3.2* in tomato (Chakrabarti et al., 2013; Frary et al., 2000). *FW2.2* is expressedin the early stages of fruit development and negatively regulates cell proliferation (Frary et al., 2000). In maize, two *FW2.2-like genes*, *ZmCNR1* (*Cell Number Regulator 1*) and *ZmCNR2*, were identified in transgenic plants (Guo et al., 2010; Guo & Simmons, 2011). Overexpression of *ZmCNR1* led to cell number changes and resulted in reduced maize ear size. *CNR/FW2.2* gene family members have also been reported in avocado, soybean and cherry, demonstrating that they play a conserved role in determining fruit size in flowering plants (Dahan et al., 2010; De Franceschi et al., 2013; Libault et al., 2010). *SlKLUH* is the gene underlying the *fw3.2* QTL and encodes a P450 enzyme of the CYB78A subfamily (Anastasiou et al., 2007). Repressing *SlKLUH* led to decreased fruit size (Chakrabarti et al., 2013). The CDK-CYC complex, which is formed by a catalytic subunit cyclin-dependent kinase (CDK) and a regulatory cyclin (CYC) subunit, determines fruit size by regulating the cell cycle (Azzi et al., 2015). In tomato *CDKA1* knockdown plants, the fruits are smaller than those of wild-type plants due to cell number loss in the exocarp of *amiCDKA1* fruits (Czerednik et al., 2012). Other identified regulators involved in cell proliferation include APETALA2/ethylene-responsive factor (AP2/ERF), growth-regulating factor (GRF), GRF-interacting factor (GIF), teosinte branched 1/cycloidea/proliferating cell factor (TCP), NAM/ATAF1/2/CUC2 (NAC), SUPERMAN, MADS-box, auxin-induced protein and auxin response factor (ARF) (Crawford et al., 2004; Horiguchi et al., 2009; Hu, Xie & Chua, 2003; Lee et al., 2009; Nibau et al., 2011; Schruff et al., 2006).

Cell expansion is another factor that controls fruit size. As the first layer of a cell, the cell wall exerts wall stress that counters cell growth. The loosening of the cell wall and the relaxation of cell wall pressure are key steps for cell expansion (Cosgrove, 2015). Multiple enzymes are involved in cell wall modification, such as expansin, pectin lyase, beta-galactosidase, xyloglucan galactosyltransferase, cellulose synthase, glycosyltransferase, microtubule-associated protein and polygalacturonase (Cosgrove, 2018; Dash, Johnson & Malladi, 2013; Hu et al., 2018; Pesquet et al., 2010; Roach et al., 2011; Scheible & Pauly, 2004). Other identified regulators that function in limiting or promoting the cell enlargement process include zinc finger protein (ZFP), basic/helix-loop-helix (bHLH), basic region/leucine zipper motif (bZIP), gibberellin-responsive protein and cytochrome P450 (Fukazawa et al., 2000; Kim et al., 1999; Schiessl et al., 2012; Yi et al., 2010).

Our previous work revealed that overexpressing an apple microRNA gene (miRNA172p) led to reduced fruit size (Yao et al., 2015). miRNAs are one of the general types of sRNAs and are the most functionally important sRNAs (small RNAs) in plants (Chen et al., 2018). For apple, a total of 146 miRNAs have been identified (Daccord et al., 2017; Ma et al., 2014). Among all identified mdo-miRNAs, miR172 is the only one that has been demonstrated to play a pivotal role in the fruit growth process (Chen et al., 2018).

There are a series of negative and positive interactions between miR172 and transcription factors from the *APETALA 2* (AP2), *ARF* and *MADS-box* families to modulate fruit growth and development (Gasser, 2015; Ripoll et al., 2015). The miR172*-AP2* module is conserved in plants. Depending on the fruit type variation , the miR172-*AP2* pathway could have different effects on fruit size (Yao et al., 2016). In *Arabidopsis*, the growth of its fruit (silique) is negatively modulated by *AP2*. Overexpression of miR172 represses the function of AP2 and breaks down AP2′s inhibition of AG (AGAMOUS) and FUL (FRUITFUL), resulting in larger siliques (Ripoll et al., 2015). In contrast, when miR172 is overexpressed in tomato, smaller-sized parthenocarpic seedless fruit are produced in an *AP2*-mediated manner (Yao et al., 2016). Similar to apple, the over-overaccumulation of miR172 led to *AP2* silencing and exhibited a dramatic reduction in fruit size and weight (Yao et al., 2015).

Currently, for perennial crops, such as apple, knowledge regarding the molecular mechanisms underlying fruit size and the transcription factors involved in the mdo-miR172-*AP2* mediated fruit growth pathway are poorly defined. In the current study, RNA-seq was performed to analyze differentially expressed genes between miR172 overexpression (miR72OX) transgenic small fruit and wild type (WT) large fruit at different developmental stages and in different tissues. The objective of this study was to identify differentially regulated genes and pathways that may underlie the observed phenotypic variations between fruits of WT and miR172OX during development. The identified DEGs will be valuable for future studies to verify their functions in fruit size determination in apple.

## Materials & Methods

### Plant materials and sample collection

Four apple genotypes, ‘Royal Gala’ (WT), ‘*35S::miRNA172p* transgenic Royal Gala’ (miR172OX), ‘Hanfu’ (*Malus x domestica*), and M9 (*Malus x domestica*), were used in this study (Yao et al., 2015). Trees of the first two genotypes were planted in Plant and Food Research (PFR, Auckland, NZ), and the remaining two genotypes were planted in Zhengzhou Fruit Research Institute, Chinese Academy of Agricultural Sciences (China, Zhengzhou). To achieve our objectives, two parallel transcriptome sequencing experiments were carried out. For the first set (172vsWT) of RNA-seq, the whole fruit (WF) at 2 WPFB (weeks post full blossom) and the fruit skin, fruit flesh and fruit core at 4 WPFB were collected from WT and miR172OX, respectively (Malladi, 2020). For the second RNA-seq dataset (M9vsHF), the whole fruit at 4 WPFB were collected from ‘Hanfu’ and ‘M9’. Three biological replicates were performed for each sample. After tissue collection, all samples were immediately frozen in liquid nitrogen and stored at −80 °C until RNA isolation.

### Total RNA isolation and library preparation for transcriptome sequencing

Total RNA was isolated as described by Zhou et al. (2018). After RNA isolation, total RNA concentration was then quantified using a Qubit^^®^^ RNA Assay Kit in a Qubit^^®^^2.0 Fluorometer (Life Technologies, Carlsbad, CA, USA), and the RNA quality was visualized on 1% agarose gels. Each tissue type or genotype was represented by three biological replicates, and every replicate included the pooled tissues from three plants. The RNA integrity number (RIN) was assessed using the Bioanalyzer 2100 system (Agilent Technologies, CA, USA). For the RNA sample preparations, 3 µg of RNA was collected per sample as input material. To generate sequencing libraries, the NEBNext^®^ UltraTM RNA Library Prep Kit for Illumina^®^ (NEB, USA) was used by following the manufacturer’s instructions. The library preparations were then sequenced on an Illumina HiSeq platform to generate 125-bp/150-bp paired-end reads.

### Sequence read mapping and differential expression analysis

Reference *Malus x domestica* GDDH13 Whole Genome v1.1 and annotation files were downloaded from GDR (https://www.rosaceae.org/) (Daccord et al., 2017). Hisat2 v2.0.5 was selected as a mapping tool to align clean reads to the reference genome (Trapnell, Pachter & Salzberg, 2009). Differential expression analysis was processed using the DESeq2 R package (1.16.1) (Benjamini & Hochberg, 1995). To control the false discovery rate, Benjamini and Hochberg’s approach was used to adjust the resulting *P* value (Benjamini & Hochberg, 1995). Differentially expressed genes (DEGs) were identified with an adjusted *P*-value (P_adj_) < 0.05 found by DESeq2 (Anders & Huber, 2010). FPKM values in at least one library greater than 2 were used as a standard to eliminate the genes with low expression.

### Validation of selected DEG expression patterns by qRT–PCR

The total RNA used for RNA-seq library construction was also used to validate RNA-seq data by qRT–PCR. Two micrograms of total RNA was used to synthesize the first strand of cDNA with the Quantitect^^®^^ Reverse Transcription Kit (Qiagen). Primers were designed with the following criteria: primer length 20–24 bp, Tm > 50 °C, GC content 45–65%, and amplicon size 150–200 bp (Table S1). Real-time qPCR amplification and detection were conducted using a Roche LightCycler 480 system (version 1.5) (Roche, Switzerland) under previously published amplification cycle conditions (Zhou et al., 2021). The target gene expression was normalized to that of a validated internal reference gene (*MD02G1221400*) using the 2^−ΔΔCT^ method (Livak & Schmittgen, 2001; Zhou et al., 2017).

### Gene coexpression network analysis

The coexpression network between structural genes and TFs was constructed based on the method described by Liu et al. (2019) and visualized in graphs by Cytoscape (Shannon et al., 2003).

## Results

### Statistical analysis of RNA-seq results from different tissues and genotypes

**Figure 1:**
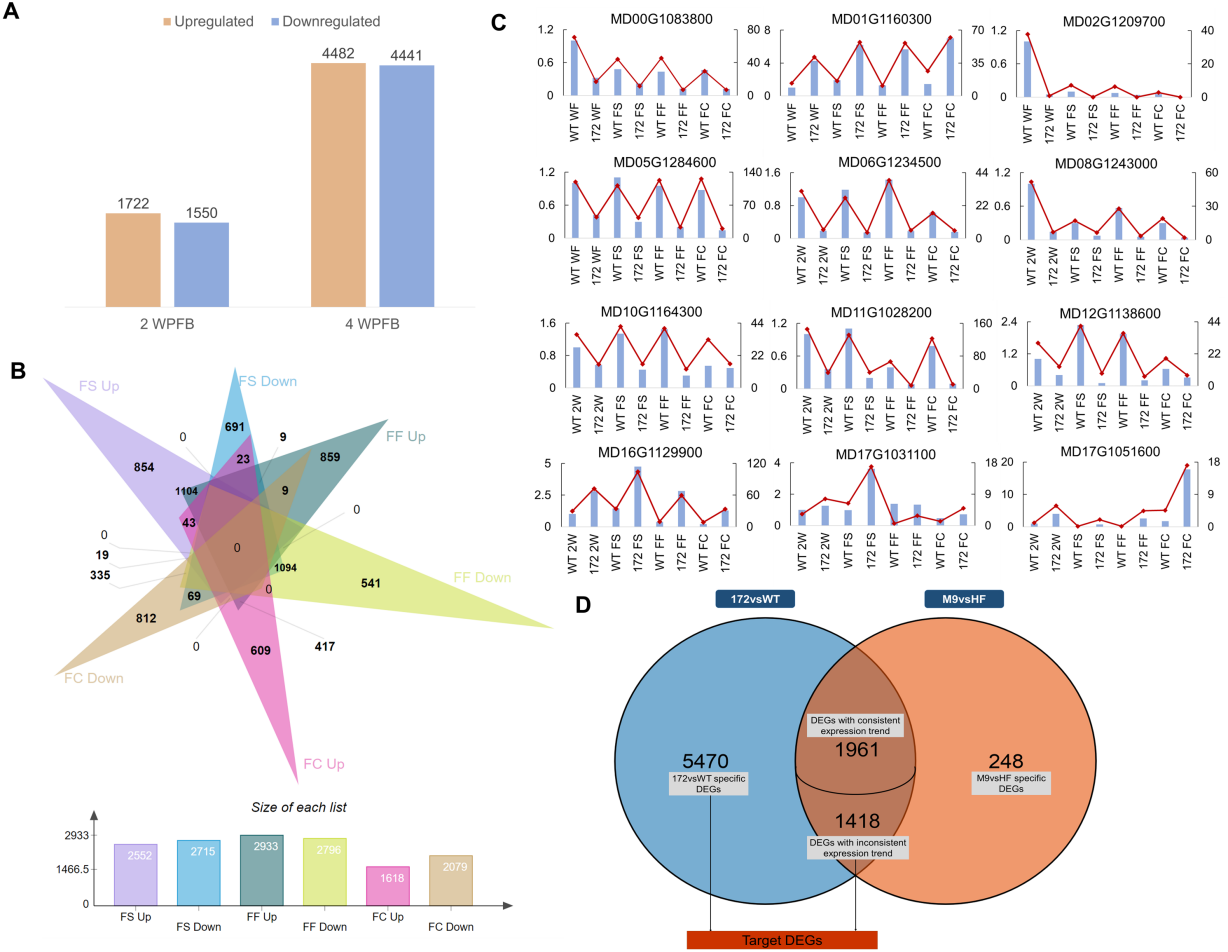
Global analysis of fruit transcriptomes in miR172OX, WT, Hanfu, and M9. (A) Bar plots showing the number of genes up-or down-regulated in miR172OX fruit tissues at 2 and 4 weeks post full bloom (WPFB) comparing to wild-type (WT) fruit. (B) A Venn diagram shows the number of genes up-or down-regulated in fruit skin (FS), fruit flesh (FF), or fruit core (FC) tissues at 4 WPFB comparing to WT fruit tissues. (C) Validation of the expression patterns for genes selected from RNA-seq analysis by qRT-PCR. The left vertical axis shows the quantitative real-time polymerase chain reaction (qRT-PCR) and the right vertical axis stands for fragments per kilo-base per million mapped reads (FPKM). (D) A venn diagram shows the number of DEGs identified between 172vsWT dataset and M9vsHF dataset.

A total of 1,473,323,002 paired-end reads of 125 bp/150 bp were generated on the Illumina HiSeq platform for 30 libraries, covering four lines (miR172OX ,WT, Hanfu and M9), three biological replicates and four tissue types, 4 WPFB fruit skin (FS), fruit flesh (FF), fruit core (FC) and 2 WPFB whole fruit (WF), for miR172 and WT, and 4 WPFB whole fruit for ‘Hanfu’ and ‘M9’. The total number of clean reads ranged from 34,240,866 to 55,998,005, with a Q20 quality score ≥ 97.28% (Table 1). Over85.92% of paired reads were mapped to the reference *Malus domestica* genome (Daccord et al., 2017). Pearson correlation analysis (*R*^2^ = 0.88 to 0.98) indicated that the three biological replicates had highly consistent transcriptome profiles across all tissue types (Fig. S1). Twelve (12) candidate genes were selected for validation with an independent qRT–PCR approach (Fig. 1C). Detailed information and the primer set for the candidate genes are listed in Table S1. Based on comparing the expression levels of miR172OX with WT, over 96% of the data points showed consistent patterns, indicating the reliability of our RNA-seq data.

**Table 1:** Summary of the RNA-seq data for four genotypes that differ in fruit size during fruit development.

Sample name	Sample description	Total reads	Q20 (%)	Q30 (%)	GC content (%)	Mapped reads	Mapping rate (%)
WF1	WT 2 WPFB whole fruit 1	41,763,828	98.04	94.08	47.76	39,382,282	94.3
WF2	WT 2 WPFB whole fruit 2	54,739,594	97.86	93.66	47.53	51,615,056	94.29
WF3	WT 2 WPFB whole fruit 3	46,224,774	97.97	93.91	47.77	43,135,917	93.32
WF4	miR172OX 2 WPFB whole fruit 1	50,289,060	97.82	93.53	47	47,087,799	93.63
WF5	miR172OX 2 WPFB whole fruit 2	43,492,410	98.07	94.08	47.14	40,934,817	94.12
WF6	miR172OX 2 WPFB whole fruit 3	44,876,944	98.03	94.02	47.32	41,910,630	93.39
FS1	WT 4 WPFB fruit skin 1	53,887,878	98.12	94.25	46.23	50,705,377	94.09
FS2	WT 4 WPFB fruit skin 2	49,229,594	97.82	93.59	46.59	46,406,775	94.27
FS3	WT 4 WPFB fruit skin 3	41,727,244	98.05	94.1	48.01	39,033,558	93.54
FS4	miR172OX 4 WPFB fruit skin 1	48,547,180	97.94	93.75	47.24	45,573,928	93.88
FS5	miR172OX 4 WPFB fruit skin 2	46,650,026	97.87	93.61	46.91	43,664,698	93.6
FS6	miR172OX 4 WPFB fruit skin 3	56,866,316	97.45	92.8	47.05	52,748,834	92.76
FF1	WT 4 WPFB fruit flesh 1	57,268,046	98.08	94.19	46.02	53,769,076	93.89
FF2	WT 4 WPFB fruit flesh 2	51,366,588	97.28	92.37	46.63	48,326,370	94.08
FF3	WT 4 WPFB fruit flesh 3	54,361,658	97.91	93.8	47.53	50,523,839	92.94
FF4	miR172OX 4 WPFB fruit flesh 1	53,349,544	98.03	93.99	46.82	50,234,774	94.16
FF5	miR172OX 4 WPFB fruit flesh 2	59,689,172	98.21	94.45	46.65	55,998,005	93.82
FF6	miR172OX 4 WPFB fruit flesh 3	54,700,608	98.06	94.18	46.58	51,233,457	93.66
FC1	WT 4 WPFB fruit core 1	52,556,132	97.81	93.56	47.35	48,904,301	93.05
FC2	WT 4 WPFB fruit core 2	42,779,592	98.13	94.28	47.27	40,442,741	94.54
FC3	WT 4 WPFB fruit core 3	43,054,320	98.11	94.27	47.83	40,052,398	93.03
FC4	miR172OX 4 WPFB fruit core 1	48,734,948	98.19	94.47	46.98	45,983,100	94.35
FC5	miR172OX 4 WPFB fruit core 2	51,990,948	98.2	94.39	46.91	49,043,759	94.33
FC6	miR172OX 4 WPFB fruit core 3	39,996,976	98.09	94.18	47.16	37,642,957	94.11
HF1	Hanfu 4 WPFB whole fruit 1	50,441,286	98.27	94.84	47.1	46,565,881	92.32
HF2	Hanfu 4 WPFB whole fruit 2	50,502,094	98.17	94.64	47.13	46,205,547	91.49
HF3	Hanfu 4 WPFB whole fruit 3	46,733,928	98.19	94.7	47.11	42,922,089	91.84
M9-1	M9 4 WPFB whole fruit 1	39,853,188	97.57	93.25	47.14	34,240,866	85.92
M9-2	M9 4 WPFB whole fruit 2	44,150,896	97.7	93.39	47.09	38,413,189	87.0
M9-3	M9 4 WPFB whole fruit 3	53,498,230	97.9	93.81	47.05	46,969,997	87.8

### Differentially expressed genes from 172vsWT and M9vsHF

Comparisons of gene expression were performed between the miR172OX samples and WT samples (P_adj_ < 0.05, Log_2_foldchange > 1 or < −1). As a result, 3,272 genes were differentially expressed at 2 WPFB, and 8849 genes were differentially expressed at 4 WPFB. A total of 1,772 of the 3,272 two-WPFB DEGs were upregulated in miR172OX and the rest were downregulated (Fig. 1A). Four-WPFB DEGs were computed on the basis of the log2-fold change of the three comparisons (172 FS *vs* WT FS, 172 FF *vs* WT FF and 172 FC *vs* WT FC). As long as one of them was >1 or <−1, that gene was considered as an upregulated or downregulated DEG. Out of the 8849 four-WPFB DEGs observed, 2,552, 2,715, 2,933, 2,796, 1,618 and 2,079 DEGs were identified as FS-upregulated, FF-upregulated, FC-upregulated, FS-downregulated, FF-downregulated and FC-downregulated genes, respectively (Fig. 1B).

**Figure 2:**
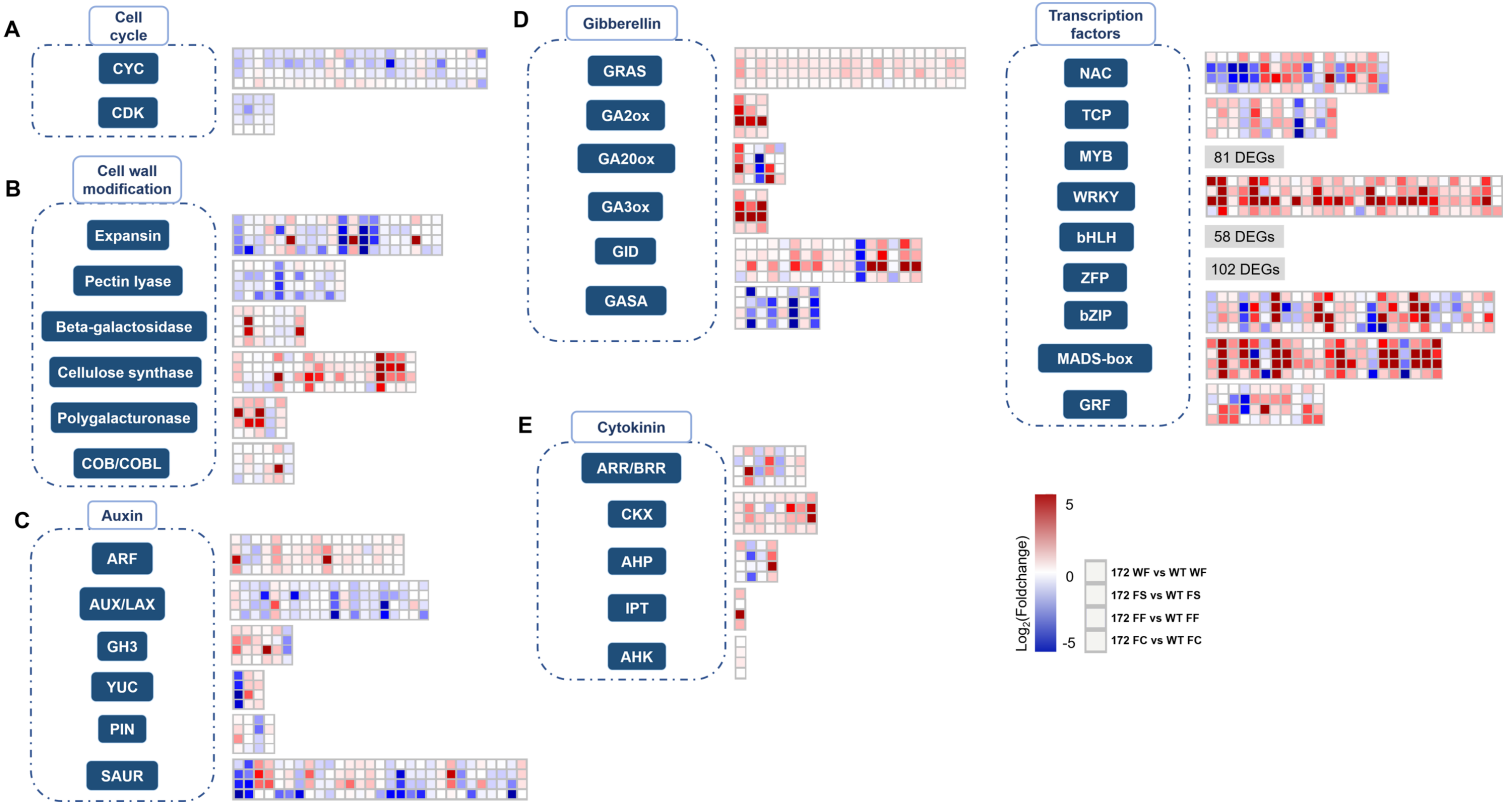
Overview of differentially expression pattern of selected genes. Heat maps depict Log_2_Foldchange values of gene expression levels between miR172OX (172) and wild-type (WT) fruit tissues. Each of four squares in a column represents for the four comparisons of one gene. Gene families with over 50 DEGs, the heat map for their Log_2_Foldchange values are not shown in this figure. (A) Cell cycle related genes. (B) Cell wall modification related genes. (C) Auxin related genes. (D) Gibberellin related genes. (E) Cytokinin related genes. (F) TF families identified from current dataset.

Based on their annotation, the identified DEGs were further screened and classified into cell cycle-, cell wall modification-, hormone-, and transcription factor (TF)-related groups (Fig. 2). To gain insight into the miR172-mediated fruit size regulatory pathways, the second set of transcriptome data regarding 4 WPFB fruits of ‘Hanfu’ and ‘M9’ was introduced. These two cultivars bear mature fruit of different weights, with average fruit weights of 250 g for ‘Hanfu’ and 75 g for ‘M9’. A total of 3,627 DEGs were detected between ‘Hanfu’ and ‘M9’. To isolate DEGs associated with miR172OX, the 4-WPFB DEGs from the two datasets were merged (Fig. 1D). As a result, 5,470 DEGs were specifically differentially expressed between miR172OX and WT. A total of 1,961 out of the 3,379 shared DEGs exhibited the same expression pattern (all upregulated or all downregulated) between non-transgenic modified and transgenic modified size variation, thus were excluded from further investigation. Similarly, the ‘M9’ *vs* ‘Hanfu’ specific DEGs were also excluded. The remaining M9vsHF DEGs and the 5470 miR172vsWT specific DEGs were then combined into one set and renamed as target DEGs for further analysis.

#### 1. Cell cycle related DEGs

The plant cell cycle is known to be essential for cell division and daughter cell generation. The key units constituting the core cell cycle machinery are CYCs (CYCLINs) and CDKs (CYCLIN-DEPENDENT KINASEs). Twenty-nine DEGs homologous to known plant core cell cycle genes were identified from 172vsWT (Fig. 2A and Table S3). Overall, 25 CYCs representing three classes (A, B and D) and 4 CDKBs were identified. An overwhelming majority, 23 out of 29 DEGs showed higher expression levels in WT samples. Among the twenty-nine 172vsWT DEGs, 16 were included in the target DEGs (Table S3). No KINASE INHIBITOR PROTEIN (KIP) or KIP RELATED PROTEIN (KRP) DEGs were observed from our RNA-seq data.

#### 2. AP2 family genes

**Figure 3:**
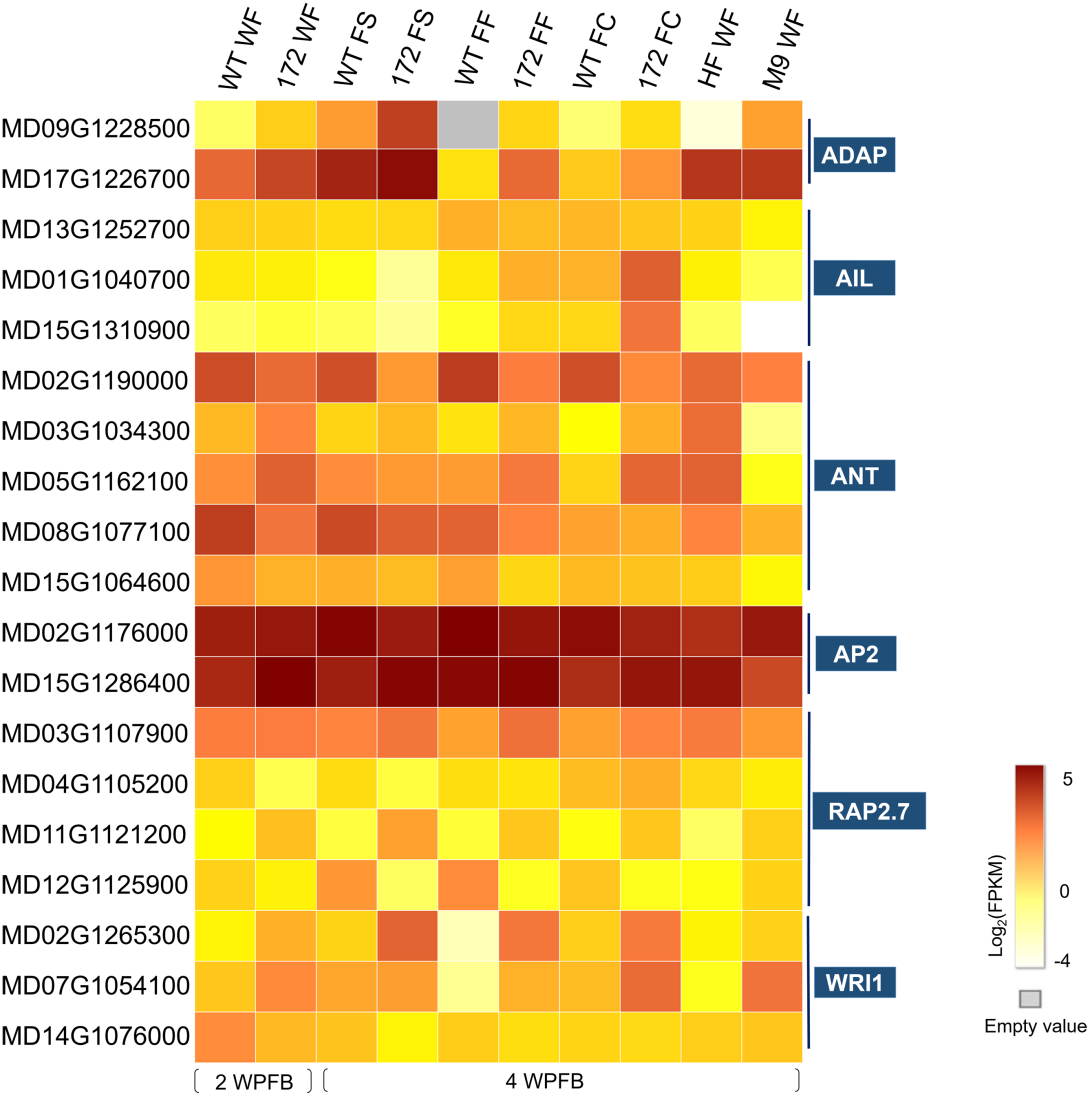
Heat map of the expression levels of selected AP2 family genes. Color scales show log2 mean FPKM value of three biological repeats of the ten tissue types that were whole fruit (WF) of wild-type (WT) and miR172OX (172) collected at 2 weeks post full bloom (WPFB), fruit skin (FS), fruit flesh (FF) and fruit core (FC) tissue of WT and miR172OX collected at 4 WPFB, and WF of ‘Hanfu’ and ‘M9’ collected at 4 WPFB. ADAP, ARIA-interacting double AP2 domain protein; ANT, AINTEGUMENTA; AIL, AINTEGUMENTA-LIKE; RAP2.7, Related to AP2.7, WRI1, WRINKLED1.

MiR172 has been demonstrated to regulate its targets through translational repression and it interacts with its *AP2* or *AP2-like* targets in a deeply conserved manner (Chen, 2004; Zhu & Helliwell, 2011). In our 172vsWT RNA-seq dataset, 114 putative *AP2* family genes were detected. Nineteen, 34 and 61 unigenes were classified into the *AP2*, *DREB* and *ERF* subfamilies, respectively (Fig. 3 and Table S2).

Among the nineteen *AP2* subfamily genes, *MD02G1176000* and *MD15G1286400* showed high expression levels in all samples. These two *MdAP2* genes shared high sequence similarity with *Arabidopsis AP2* and were demonstrated to be potential targets for *miRNA172p* by bioinformatics analysis (Yao et al., 2015). In addition, *ANT* (*AINTEGUMENTA*) and *AIL* (*AINTEGUMENTA-LIKE*) within the *AP2* subfamily have also been implicated in regulating fruit growth (Dash & Malladi, 2012). In the 172vsWT dataset, 7 *ANT/AIL* genes were observed, including 4 *ANT*s and 3 *AIL*s. All *ANT/AIL* genes except one (*MD13G1252700*) were also be found in the target DEGs (Table S2).

#### 3. DEGs encoding enzymes involved in cell wall modification

Cell expansion requires cell wall alteration. In this study, DEGs encoding cellulose synthase (CS), pectin lyase, beta-galactosidase, expansin (EXP) and polygalacturonase (PG) were identified between miR172OX and WT (Fig. 2B and Table S3). Cellulose synthase impacts cell wall integrity and cell growth which eventually contribute to fruit growth (Hu et al., 2018). The majority of identified CS DEGs in this study showed higher expression levels in miR172OX. Approximately six times more CS DEGs were expressed at a higher level in miR172OX-4 WPFB than in WT-4 WPFB. Beta-galactosidase, pectin lyase, expansin and PG are cell wall degradation enzymes that promote cell separation and increase cell size (Aro, Pakula & Penttila, 2005; Cosgrove, 2015; Marin-Rodriguez, Orchard & Seymour, 2002). Almost all identified expansin-encoding DEGs were downregulated in miR172OX, with a few exceptions. Four out of seven identified beta-galactosidase-encoding DEGs were upregulated in miR172OX-libraries. Two genes, *MD02G1079200* and *MD15G1206800*, consistently had higher expression level in miR172OX from two WPFBs to four WPFBs in all tissue types. For PG-encoding DEGs identified from the present dataset, all but one (*MD16G1092600*) was downregulated in the miR172OX tissues compared with the respective WT tissues. Notably, *MD16G1092600* transcript levels were at least 11 times higher than those of other PG-DEGs. All identified pectin lyase encoding DEGs were 4WPFB downregulated DEGs. Notably, apart from the common downregulation at 4WPFB for all pectin lyase-encoding DEGs, *MD13G1106200* and *MD16G1078000*, upregulation was also obeserved at 2 WPFB. They both exhibited high expression levels of miR172OX at 2 WPFB, but their expression decreased dramatically at 4 WPFB. However, their expression patterns in WT were the opposite. Additionally, a set of *COB* (*COBRA*) and *COBL* (*COBRA-LIKE*) family DEGs were also observed from our 172vsWT RNA-seq dataset. One *COB* gene (*MD06G1203700*) and one *COBL* gene (*MD14G1214900*) were down- and upregulated, respectively. Their expression patterns are consistent with previous studies (Dash, Johnson & Malladi, 2012; Dash, Johnson & Malladi, 2013).

#### 4. Hormone-related DEGs

**Figure 4:**
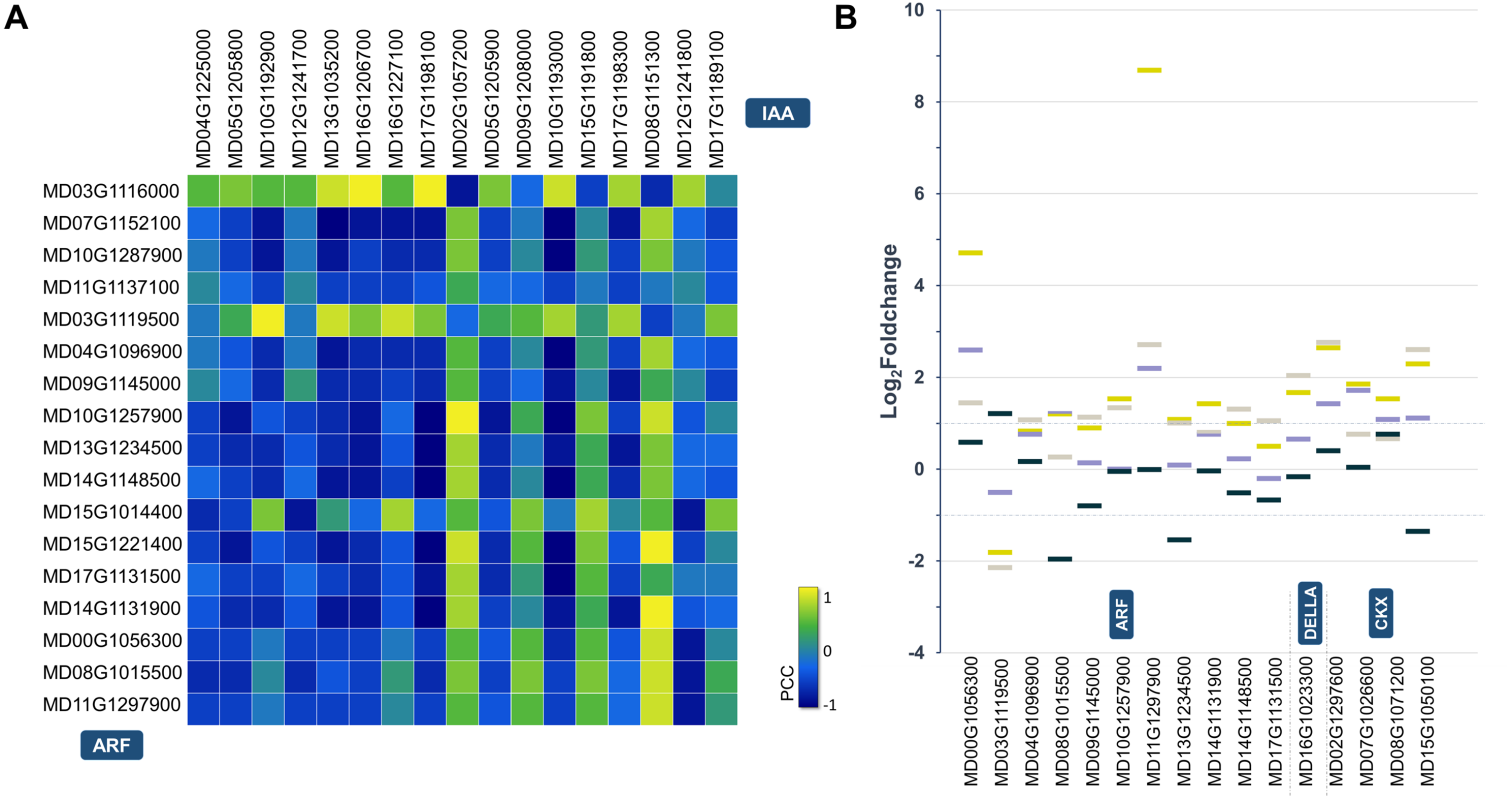
Statistical analysis of the hormone-related DEGs. (A) Identifying protein interactions between spatiotemporally co-expressed ARF (auxin response factor) and Aux/IAA (auxin-responsive protein). Heat map showing Pearson correlation coefficient (PCC) scores between auxin signaling related *ARF* and *Aux/IAA* genes. (B) Expression pattern difference of hormone-related target DEGs. A scatter diagram depict Log_2_Foldchange values produced by 172vsWT and M9vsHF at 4WPFB. ARF auxin response factor, CKX cytokinin oxidase.

The conserved miR172-*AP2* regulatory module is the bridge that coordinates the morphogenesis and hormonal pathways to control fruit size (Ripoll et al., 2015). In the current study, a total of 76 auxin-related DEGs between WT and miR172OX were detected, including 17 ARFs, 3 YUCCAs (YUCs), 6 GH3s, 4 PINs, 29 SAUR-like auxin-responsive proteins and 20 auxin-resistant 1/Like AUX1s (AUXs/LAXs) (Fig. 2C and Table S3). Auxin response factors (ARFs) and auxin/IAA (Aux/IAA) are two related groups of proteins, and different ARF-Aux/IAA modules are known as key regulators of auxin-modulated gene expression and different developmental processes (Liscum & Reed, 2002; Vanneste & Friml, 2009; Xu et al., 2019). Hence, a pairwise gene expression correlation analysis between 17 ARF DEGs and 14 Aux/IAA DEGs identified from 172vsWT was performed (Fig. 4A). After applying a cutoff (>0.90/<−0.90 for positive/negative correlation) to the correlation coefficient, 6 potential ARF-Aux/IAA combinations were found, including MdARF4 (MD03G1116000)-MdIAA14 (MD16G1206700) (Pearson correlation coefficient (PCC) = 0.96), MdARF4 (MD03G1116000)-MdIAA19 (MD17G1198100) (PCC = 0.97), MdARF16 (MD04G1096900)-MdIAA22D (MD10G1193000) (PCC = −0.91), MdARF6 (MD10G1257900)-MdIAA19 (MD17G1198100) (PCC = −0.94), MdARF9 (MD14G1131900)-MdIAA29 (MD08G1151300) (PCC = 0.93), and MdARF2 (MD14G1148500)-MdIAA22D (MD10G1193000) (PCC = −0.93).

DEGs encoding enzymes and proteins functioning in gibberellin biosynthesis, degradation and signaling are listed in Fig. 2D and Table S3. These enzymes and proteins include 3 gibberellin 2-oxidases (GA2oxs), 5 GA20oxs, 3 GA3oxs, 17 gibberellin-insensitive dwarf protein 1 (GID1), 22 GRASs and 8 GASA family proteins (Ogawa et al., 2003). Five gene families encoding proteins that were involved in cytokinin metabolism (biosynthesis, conjugation and degradation) and signal transduction, including isopentenyltransferase (IPT), cytokinin oxidase (CKX), A/B-type response regulator (ARR/BRR), AHK (histidine kinase) and AHP (histidine phosphotransfer protein), were observed in the miR172OX *vs* WT-DEGs (Fig. 3E and Table S3).

However, despite the wealth of accumulated 172vsWT DEGs, it is still not very clear how the regulation of fruit growth by the coordination of miR172-AP2 with hormone-related genes is achieved mechanistically. Therefore, gene families that have been reported to be involved in miR172 mediated hormone signaling including ARF, DELLA, and CKX were selected and then purposefully narrowed down through target DEGs (Aguilar-Jaramillo et al., 2019; Di Marzo et al., 2020; Ripoll et al., 2015; Yu et al., 2012). Eleven, 1, and 4 target DEGs encoding ARFs, DELLA, and CKXs were finalized (Fig. 4B).

#### 5. DEGs encoding TFs

In our study, in addition to the TFs mentioned above, there were 365 more genes encoding nine major TF families potentially involved in fruit size regulation network were altered in miR172OX (Fig. 2F and Table S3). Seventeen, 12, 81, 29, 58, 102, 30, 22 and 11 genes were annotated to *NAC*, *TCP*, *v-myb avian myeloblastosis viral oncogene homolog* (*MYB*), *WRKY*, *bHLH*, *ZFP*, *bZIP*, *MADS box* and *GRF*. For TFs, more DEGs were upregulated in miR172OX.

### Expression analysis of DEGs involved in the phenylpropanoid pathway

**Figure 5:**
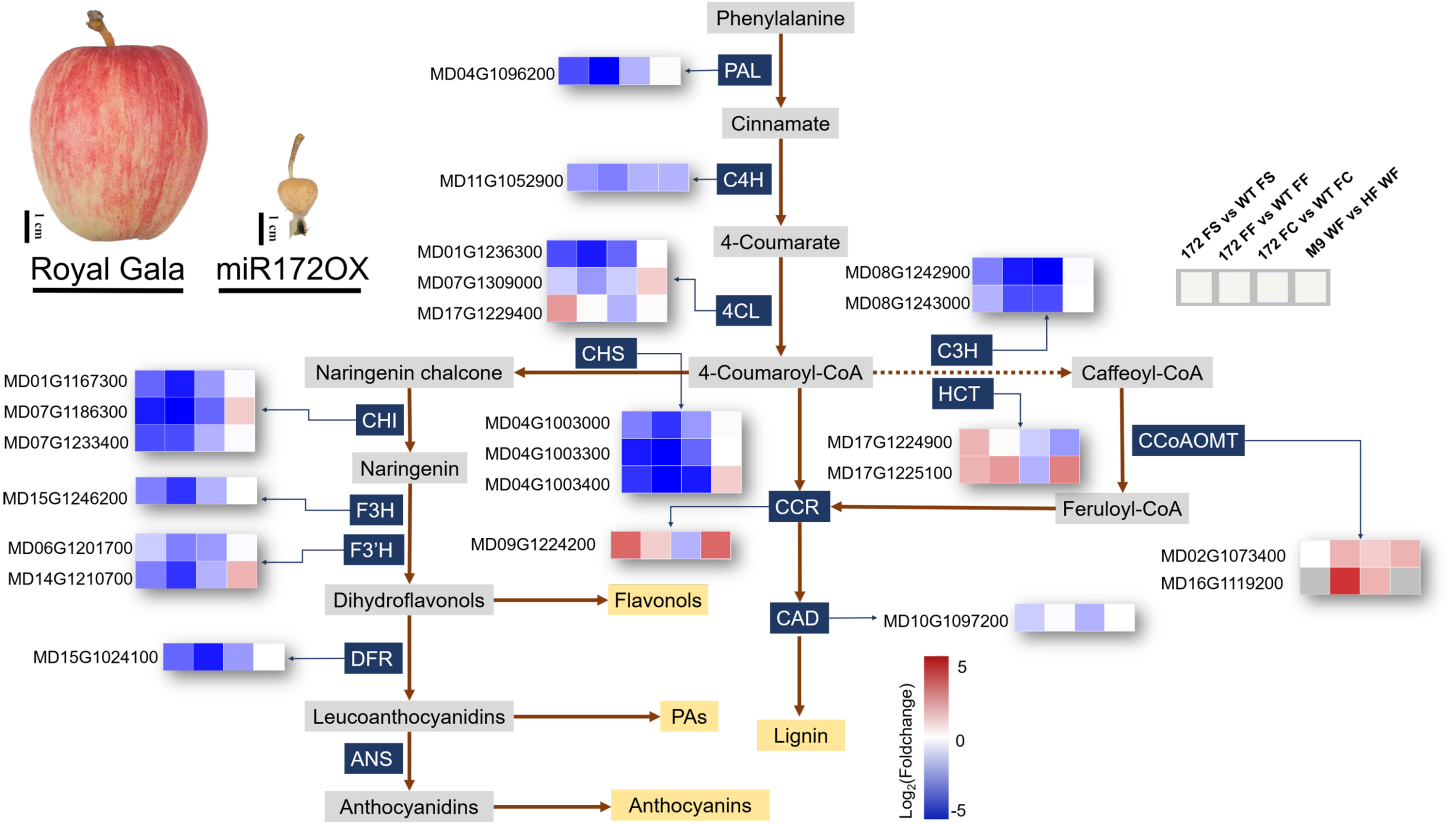
Simplified scheme and heat maps of the differentially expressed genes (DEGs) involved in phenylpropanoid pathways. Phenotypes showing fruits from ‘Royal Gala’ control and ‘35S::miRNA172p transgenic Royal Gala’ (miR172OX). Heat maps depict Log2Foldchange values of gene expression levels ofmiR172OX (172) *vs* wildtype (WT) fruit tissues, and ‘M9’ *vs* ‘Hanfu’. Each of four squares in a row represents for the four comparisons of one gene. 4CL 4-coumarate:CoA ligase, C3H p-coumarate 3-hydroxylase, C4H Cinnamate 4- hydroxylase, CCR Cinnamoyl CoA reductase, F3H Flavonone-3-hydroxylase, F3′H Flavonoid 3- monooxygenase, CAD Cinnamyl alcohol dehydrogenase, HCT Hydroxycinnamoyltransferase, PAL Phenylalanine ammonia lyase, CHS Chalcone synthase, ANS Ferulate 5-hydroxylase, DFR Dihydroflavonol 4-reductase, CCoAOMT Caffeoyl-CoA 3-O-methyltransferase.

Fruit size is known to have a strong correlation with the composition of secondary metabolites (Bakir et al., 2020). Apart from miR172OX bearing smaller fruits, overexpressing miR172p also resulted in wrinkled and rough fruit peel (Fig. 5). Therefore, it is highly possible that miR172 may affect the phenylpropanoid pathway by directly or indirectly regulating pathway node genes.

#### 1. DEGs encoding enzymes of the phenylpropanoid pathway

The expression level of secondary metabolites during fruit growth and development are closely related to special gene expression. According to the KEGG pathway analysis, in our 172vsWT RNA-seq data, 23 phenylpropanoid biosynthesis related enzymatic DEGs were identified and their regulation patterns further exemplify the contrasting transcriptome changes between WT and miR172OX during the early stage of fruit development (Fig. 5). DEGs encoding enzymes functioning in the flavonoid pathway were systematically downregulated in miR172OX. These enzymes include chalcone synthase (CHS), chalcone isomerase (CHI), flavonone-3-hydroxylase (F3H), flavonoid 3-monooxygenase (F3′H), and dihydroflavonol-4-reductase (DFR). The three DEGs encoding 4-coumarate: coA ligase (4CL) could be grouped into two clusters, Class I (*MD17G1229400*) and Class II (*MD01G1236300* and *MD07G1309000*), based on phylogenetic analysis with known 4CLs from other species (Fig. S2). The Class I cluster is mainly responsible for monolignol biosynthesis, whereas Class II is involved in flavonoid biosynthesis other than lignin (Lavhale, Kalunke & Giri, 2018). Consistent with the regulation pattern of other flavonoid biosynthesis related DEGs, *MD01G1236300* and *MD07G1309000* exhibited higher expression levels in WT. Several gene families encoding enzymes that catalyze lignin biosynthesis, including Class I 4CL, hydroxycinnamoyltransferase (HCT), and cinnamoyl CoA reductase (CCR) showed higher expression levels in miR172OX-FS. Twenty-two out of 23 of the 172vsWT DEGs showed potential miR172 regulatory dependence after comparison with M9vsHF DEGs (Fig. 5 and Table S4).

#### 2. Coexpression networks between phenylpropanoid pathway structural genes and TFs

**Figure 6:**
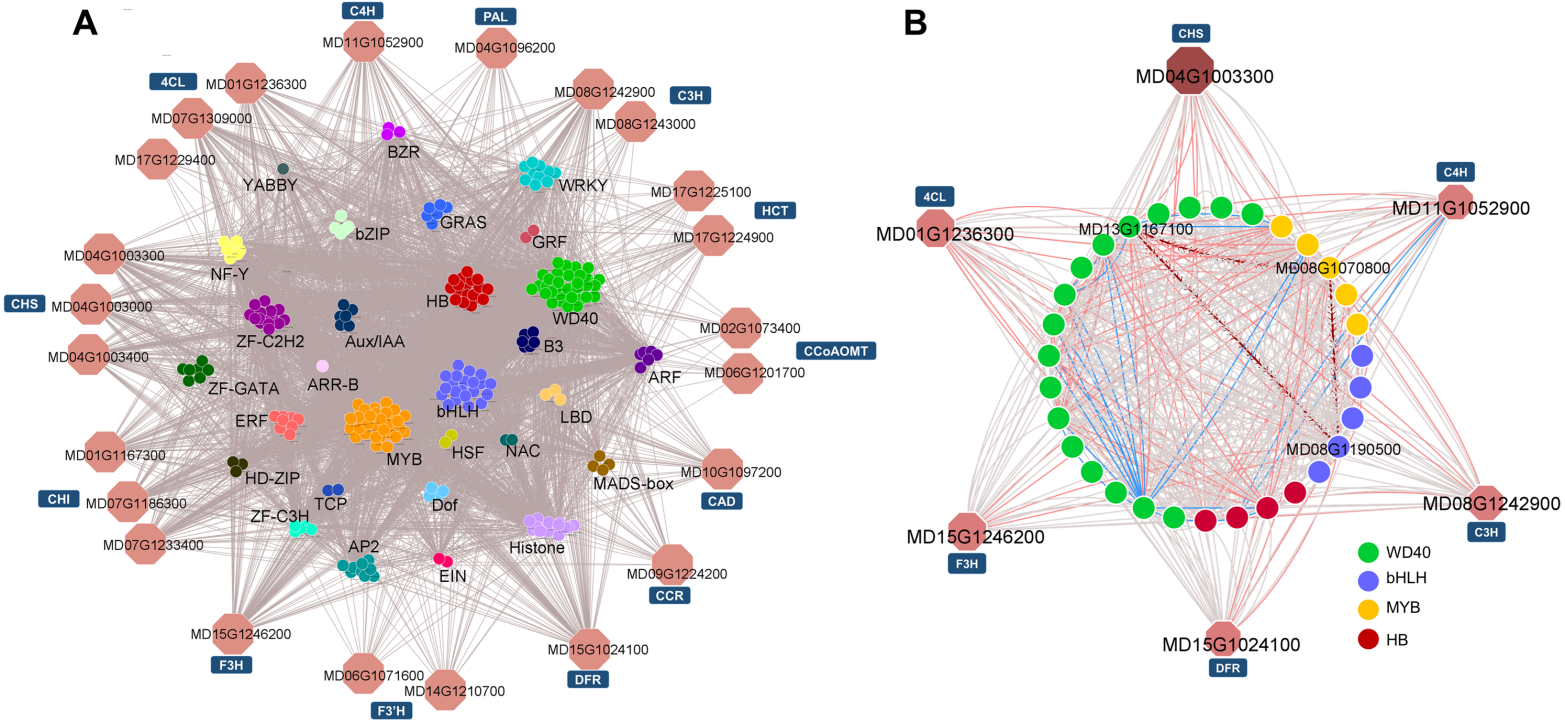
Potential regulatory networks involved in phenylpropanoid pathways. (A) Sub-network for phenylpropanoid biosynthesis. (B) *MdCHS-2*-guided sub-network. The red and blue edges represent edges that possess a cutoff correlation coefficient over 0.95 or less than −0.95. Structural genes identified as co-expressed with *MdCHS-2* (*MD04G1003300*) are shown in red.

Based on the Pearson product-moment correlation coefficient (PPMCC), the coexpression networks between structural genes and TFs were constructed using the transcript profile data (cutoff-value: >0.9/<−0.8 for positive/negative correlation)(Liu et al., 2019). According to the KEGG pathway analysis, 20 phenylpropanoid biosynthesis-related enzymatic genes and their potential direct regulatory TFs were extracted for subnetwork analysis (Fig. 6A). Among the 361 TFs, *WD40* family members, *MYB* family members and *bHLH* family members were the top three most numerous. Members from these three families are known to form a ternary protein complex (MYB-bHLH-WD40, MBW) and tightly control the expression of flavonoid and lignin biosynthetic genes (Fornale et al., 2010; Guo et al., 2017; Wang et al., 2019).

*MdCHS-2* has been demonstrated to play a significant physiological role in fruit development as *MdCHS-2*-silenced lines produce smaller fruits (Dare et al., 2013). Therefore, to further clarify the detailed TF-structure gene regulatory networks that are responsible for the alteration of the phenylpropanoid biosynthetic pathway between WT and miR172OX, a guide-gene approach employing *MdCHS-2* (*MD04G1003300*) was used to identify coexpressing genes (Shinozaki et al., 2018). The top ten positively and negatively coexpressed genes with *MD04G1003300* were screened out and combined into a subnetwork (Fig. 6B). The subnetwork included five other core genes encoding enzymes (4CL, F3H, DFR, C3H, and C4H) in the flavonoid biosynthetic pathway and TFs from the WD40, MYB, bHLH and homeobox families (edges between structural genes are not shown). After applying a very stringent cutoff (±0.95) for the correlation coefficient (edges highlighted in red and blue) to the *MdCHS-2*-guided subnetwork, an MBW (*MD08G1070800*, *MD08G1190500* and *MD13G1167100*) molecule (dark red contiguous arrow connected nodes) potentially associated with flavonoid pathways was revealed (Fig. 6B).

## Discussion

The current understanding of the molecular mechanism of fruit size, particularly the fruit size of perennial tree crops, is very limited. In the present study, high-throughput sequencing technology was employed to carry out RNA-seq-based transcriptome analysis of the previously reported miR172 overexpression line in apple (Yao et al., 2015). Our previous research showed that, as early as 2 WPFB, cell number loss can be observed in miR172OX fruit in comparison with WT fruit. For cell size inhibition, miR172 did not exhibit its inhibitory effect until late stages of fruit development. Therefore, two time points, 2 WPFB and 4 WPFB were chosen as sampling times to explore key DEGs involved in the processes of cell proliferation and cell expansion.

Cell proliferation is accomplished by a cell sequentially going through different phases of the cell cycle (Malladi & Johnson, 2011). The plant mitotic cell cycle consists of G1 (cell growth), S (DNA replication), G2 (DNA repair) and M (mitosis occur) phases (Inze & De Veylder, 2006). B-type CDKs and A- and B-type CYCs, which constituted G2/M phase cell cycle regulators, accounted for over two-thirds of the identified CYCs and CDKs in our RNA-seq data (Table S4). This indicates that the availability of putative G2/M phase cell cycle regulators could be a limiting factor for cell proliferation during fruit development. In addition, the majority of the CDKBs, CYCAs and CYCBs encoding DEGs showed downregulation in miR172OX fruit, suggesting that they are likely to be positively associated with cell proliferation (Fig. 2A). Among all D-type CYCs, CYCD3;1 has been demonstrated to be a switch for cells to transition from cell proliferation to final stage differentiation (Dewitte et al., 2003; Menges et al., 2006). *MD05G1087300* was a highly expressed DEG annotated to CYCD3;1. Contrary to the overall downregulation trend of CYCs, *MD05G1087300* showed higher expression levels in miR172OX fruits. This is the same as in *Arabidopsis*, where overexpressing *CYCD3;1* led to smaller organs (Dewitte et al., 2003). The increased expression levels of *MD05G1087300* in miR172OX fruits are likely to follow the regulation rules in *Arabidopsis*, which interfere with the cell cycle causing cells to fail to differentiate and expand normally.

Upstream of cell cycle genes, there are a series of transcription factors that can modulate their transcript abundance. One such transcription factor is *ANT* within the AP2 domain family (Horiguchi et al., 2009; Mizukami & Fischer, 2000). ANT stimulates growth by proliferation as *ANT-* overexpressing plants have larger organs formed by more cells (Mizukami & Fischer, 2000). Two of the four ANT-encoded target DEGs were also identified as *Arabidopsis* ANT homologs in apple, *MD02G1190000* and *MD15G1064600*, exhibited downregulation of expression possibly caused by the overexpression of miR172. The other two ANTs (*MD03G1034300* and *MD05G1162100*) encoding target DEGs showed opposite expression patterns in 172vsWT compared with M9vsHF (Fig. 3). *Arabidopsis* ANT is a transcriptional activator that plays a positive role in regulating the expression of *CYCD3;1* (Mizukami & Fischer, 2000). Since both the *MdANTs* (MD*03G1034300* and *MD05G1162100*) and *MdCYCD3;1* (*MD05G1087300*) showed mi172-related downregulation, it is likely that the mechanism by which *ANTs* participate in fruit development regulation by stimulating *CYCD* genes also exists in apple and is affected by miR172. Other members of the *ANT* subfamily, such as *AIL6*, are also involved in modulating proliferation and show similar effects on growth through proliferation as ANT (Krizek, 2009; Nole-Wilson, Tranby & Krizek, 2005). *AIL6s* in apple have been reported to display a sharp expression decline between flower development and early fruit development, and continue to remain at low levels throughout the rest of fruit development (Dash & Malladi, 2012). Two AIL6-encoding DEGs, *MD01G1040700* and *MD15G1310900* only demonstrated dramatic upregulation in the miR172OX fruit core at 4 WPFB (Fig. 3). Therefore, it is proposed that ANTs and AILs are important components of the miR172-mediated developmental network that regulates the extent of cell division and thereby controls fruit growth in apple. Eight potential AP2-like targets of miRNA172p in apple were reported in our previous study, while six of these eight were expressed in current RNA-seq data. It has been well demonstrated that, in *Arabidopsis*, miRNA172 exerts its function on AP2 by suppressing AP2 translation without affecting AP2 transcript abundance (Chen, 2004). Therefore, the strongly expressed and AtAP2 closely related AP2-encoding genes, *MD02G1176000* and *MD15G1286400*, are most likely to be involved in the miRNA172p-mediated fruit size modification pathway.

The arrest of proliferation is followed by another growth phase in which the fruit grows by cell expansion, driven by relaxation of the cell walls. Unsurprisingly, many cell wall-modifying proteins appeared to be functional during fruit development as they were identified as DEGs (Fig. 2B). Proteins from the expansin family are the key component of cell wall loosening and a new cell wall synthesis process is required for cell expansion. Since overexpression of *expansins* results in increased organs with larger cells in *Arabidopsis* and the majority of expansin encoding DEGs in our dataset had higher expression levels in WT, the low transcriptional abundance of *expansins* is one of the limiting factors for growth by expansion in miR172OX fruits. Pectin is another major component of the plant cell wall and has functions including involvement in maintaining plant growth. Pectin lyase and PG are the two classes of pectin-degrading enzymes (Hongo et al., 2012; Wolf, Mouille & Pelloux, 2009). From the current dataset, DEGs annotated to pectin lyase were exclusively downregulated in miR172OX-4WPFB. For PG-encoding DEGs, a single and the only upregulated *PG* transcript (*MD16G1092600*) accounted for a large proportion of the total identified *PG* transcripts, suggesting that pectin lyases and PGs play positive roles in regulating fruit size. However, unlike pectin lyases, none of the PG-encoding genes were identified as target DEGs, suggesting that PGs affect fruit size in a miR172-independent manner. The exact roles of cell wall modification genes in modulating apple fruit growth are unclear, and a systematic functional analysis of these genes mentioned above is necessary to determine the main components related to fruit growth-associated cell wall loosening.

To coordinate cell proliferation and expansion, hormones are essential factors and exert profound effects on fruit growth. Among various hormones, auxin is of the most importance to cell proliferation and expansion (Pei et al., 2020). Six groups of auxin-related genes encoding putative responsive proteins, signaling proteins and transport proteins were observed to be significantly altered in this dataset (Fig. 2C). One of these groups is the ARF family, members of which have exhibited direct activation of the expression of the miR172-encoding gene to enhance fruit growth (Ripoll et al., 2015). In additon, ARF and Aux/IAA could interact with each other and form an integral part of the auxin signaling pathway. In apple, MdARF13 interacts with its repressor, MdIAA121, to regulate anthocyanin biosynthesis in an auxin signal mediated manner (Wang et al., 2018). Six other pairs of ARF-Aux/IAA combinations were discovered in our study based on the RNA-seq data (Fig. 4A). Among these, except two interacting combinations formed with MdARF4 (MD03G1116000) that did not seem to be regulated by miR172, the remaining candidate pairs potentially participated in the regulation of apple fruit development by mediating miR172 signal transduction. ARF6 has been shown to be involved in the miR172-mediated pathway to regulate fruit development in *Arabidopsis*. Here, in our dataset, MdARF6 (MD10G1257900) and its potential interacting patterner, MdIAA19 (MD17G1198100), were discovered. As the closest ortholog of AtARF6 in apple, MdARF6 is likely to play a similar role. Moreover, we offered a method of how transcriptome data could be used to predict protein interactions in perennial fruit trees, such as apple. Our RNA-seq data indicated a detailed apple fruit growth regulatory network involving auxin-related genes. possibly adding evidence to the impact of auxin on fruit quality traits in the future.

The role of cytokinins in controlling cell proliferation and cell division is a defining characteristic of this phytohormone (Schaller, Street & Kieber, 2014). Twenty-one CK-related DEGs were identified from our dataset, including eight CKX encoding DEGs. CKX is involved in the metabolic degradation of cytokinins. In *Arabidopsis*, *ckx* mutants form lagerer floral meristems and inflorescences. At the same time, the released high activity of the *ckx* placenta develops supernumerary ovules, resulting in an increase in seed set per silique (Bartrina et al., 2011). In our dataset, two CKX7s (*MD08G1071200* and *MD15G1050100*) encoding target DEGs all had significantly higher expression levels of miR172OX at 4 WPFB in cortex and core tissues. *AtCKX7* has been reported to be directly positively regulated by STK, which is required in the miR172 signaling pathway, to control fruit size (Di Marzo et al., 2020)). The *ckx7* mutants possess shorter fruits than the wild type. In the present work, the expression profile of CKX7s (*MD08G1071200* and *MD15G1050100*) was highly consistent with two identified STK-encoding DEGs (*MD08G1216500* and *MD15G1403600*), especially in fruit flesh and fruit core (Table S3), suggesting that apple STK may also influence fruit expansion by regulating cytokinin degradation *via* interaction with CKX7. GA catabolic enzyme-encoded genes such as GA20oxs and GA2oxs were noticeably upregulated in miR172OX (Fig. 2D). Almost all of the GA20oxs and GA2oxs showed drastic changes between WT and miR172OX at 4 WPFB in fruit flesh. Since overexpressing GA20ox and GA2ox in tomatoes has been reported to promote growth and lead to the production of larger organs (Garcia-Hurtado et al., 2012), it is proposed that these genes are more likely to play delayed roles in cell proliferation due to 35S:miR172 interference. Important GA-responsive genes, such as GASAs, were downregulated in miR172OX, and thus contributed to reduced fruit size.

During fruit development, coordinated genetic and biochemical events take place, which could result in changes to fruit color, flesh texture and flavor (Alexander & Grierson, 2002; Fraser et al., 2007; Giovannoni, 2004). These developmental changes and fruit quality traits are tightly related to secondary metabolite contents in apple fruits. In the present study, genome-wide identification of the coexpressing genes of *MdCHS-2* may suggest that the phenylpropanoid pathway may regulate diverse fruit developmental processes. The resolved pathways revealed that an MBW complex composed of TFs from the MYB (*MD08G1070800*), bHLH (*Md08G190500*) and WD40 (*MD13G1167100*) families collectively regulates phenylpropanoid biosynthesis in apple fruit tissues. *MD08G1070800* is homologous to *AtMYB12,* which has been reported to be a strong activator of the promoters of *CHS*, *F3H* and *FLS*. In maize, *ZmC* 1, as *MD08G1070800*’s homologous gene, combines with the bHLH factor ZmSn to positively activate the promoter of *DFR* (Mehrtens et al., 2005). Similar target gene specificity could also be observed in the current study. Since R2R3-MYB factors are highly conserved among plant species, *MD08G1070800* is likely to mediate the phenylpropanoid pathway by activating target promoters independently or dependently of the presence of bHLHs. In addition, being one of the key branching point enzymes and the last of the three shared common enzymes in the general phenylpropanoid pathway, 4CL contributes to channelizing precursors for different phenylpropanoids (Lavhale, Kalunke & Giri, 2018). In loquat fruit, EjAP2-1 could exert an effect on the *Ej4CL1* promoter through interaction with *EjMYB* transcription factors to affect phenylpropanoid pathway metabolites (Zeng et al., 2015). The overall expression level fluctuation of the *Md4CLs* between the two genotypes may also be attributed to the alteration of AP2s caused by overexpressing miRNA172p, indicating that a regulatory mechanism similar to that of loquat may also exist in apple.

In the process of annotating DEGs, we discovered that many reported fruit developmental-associated TF families, as well as hormone biosynthesis and signal transduction related genes, did not exhibit a unique expression pattern. For example, genes within the same families or included in the same regulatory pathways were not simultaneously up- or downregulated. Multiple regulation schemes may exist. For example, miR172 selectively regulates specific members of the AP2 family or members from other gene families. Similar regulatory mechanisms have been reported for miR156, which targets part of the *SQUAMOSA promoter-binding protein like* (*SPL*) family (Rhoades et al., 2002). Moreover, TFs can be divided into transcriptional activators and repressors that either turn on or turn off a gene’s transcription based on their functional roles, and feedback regulation exists for most TFs, which could also contribute to totally opposite expression patterns for TFs from the same family. The third possible reason for irregular TF expression patterns could be attributed to miR172. Instead of having an impact on the entire fruit developmental regulatory network, miR172 is more likely to indirectly affect specific pathways, and only a small number of genes have regulatory roles in fruit development. The current study indicates that the effect of miRNA172p overexpression in Royal Gala fruit is complex and profound. Overexpressing miRNA172p appears to finalize (*i.e.,* impact) fruit size by affecting the cell cycle, phytohormones, cell wall and metabolism. The observed transcriptional changes appear to be concentrated in the regions related to fruit growth, such as core and cortex tissues. The results from our study provide a solid foundation for future hypothesis-driven validations of these candidate genes with fruit developmental traits.

## Conclusion

The overall understanding of the fruit size regulatory mechanism in apple is limited. This communication helps unravel the global transcriptional networks of apple fruit early-stage development that are dependent on or independent of miR172. The dataset generated from this comparative transcriptome analysis showed contrasting scenarios between WT and miR172OX. The more dramatically changed transcriptomes in the fruit of miR172OX reflected a larger number of DEGs at 2 and 4 WPFB, critical stages for fruit enlargement. Furthermore, a majority of the 172vsWT DEGs exhibited miR172-specific changes in expression profiles. Such genes included those encoding AP2s, cell wall modification enzymes, cell cycle-related proteins, hormone-related family members, TFs, and secondary metabolite biosynthesis. These observations indicated that some of these genes are likely to be direct targets of miR172, such as AP2s, and some are potentially involved in miR172-mediated pathways. Thus, here we provide an infrastructure that could offer a source for future identification of these casual genes. Regulatory genes, including those in the hormone biosynthetic and signaling pathways with high miR172-associated expression patterns were also analyzed. Auxin, GA, and CK were implicated in affecting fruit size under the control of miR172 through the expression dynamics of these genes. Systematic suppression of the secondary metabolism in miR172OX, including flavanols and many other metabolic pathways, seemed to disrupt cell division and enlargement at early fruit growing stages. The identified DEGs from the current study offer a valuable source of candidate genes for elucidating their association with fruit size traits with or without the involvement of miR172.

## Supplemental Information

10.7717/peerj.12675/supp-1 Supplemental Information 1 Supplementary tables

10.7717/peerj.12675/supp-2 Supplemental Information 2 RT-PCR raw data

10.7717/peerj.12675/supp-3 Supplemental Information 3 Supplementary figures

## Additional Information and Declarations

### Data Availability

The following information was supplied regarding data availability: The sequencing reads are available at SRA: PRJNA649660.

## References

[ref-1] Aguilar-Jaramillo AE, Marin-Gonzalez E, Matias-Hernandez L, Osnato M, Pelaz S, Suarez-Lopez P (2019). TEMPRANILLO is a direct repressor of the microRNA miR172. The Plant Journal.

[ref-2] Alexander L, Grierson D (2002). Ethylene biosynthesis and action in tomato: a model for climacteric fruit ripening. Journal of Experimental Botany.

[ref-3] Anastasiou E, Kenz S, Gerstung M, MacLean D, Timmer J, Fleck C, Lenhard M (2007). Control of plant organ size by KLUH/CYP78A5-dependent intercellular signaling. Developmental Cell.

[ref-4] Anders S, Huber W (2010). Differential expression analysis for sequence count data. Genome Biology.

[ref-5] Aro N, Pakula T, Penttila M (2005). Transcriptional regulation of plant cell wall degradation by filamentous fungi. Fems Microbiology Reviews.

[ref-6] Azzi L, Deluche C, Gevaudant F, Frangne N, Delmas F, Hernould M, Chevalier C (2015). Fruit growth-related genes in tomato. Journal of Experimental Botany.

[ref-7] Bakir S, Capanoglu E, Hall RD, De Vos RCH (2020). Variation in secondary metabolites in a unique set of tomato accessions collected in Turkey. Food Chemistry.

[ref-8] Bartrina I, Otto E, Strnad M, Werner T, Schmulling T (2011). Cytokinin regulates the activity of reproductive meristems, flower organ size, ovule formation, and thus seed yield in Arabidopsis thaliana. The Plant Cell.

[ref-9] Benjamini Y, Hochberg Y (1995). Controlling the false discovery rate—a practical and powerful approach to multiple testing. Journal of the Royal Statistical Society Series B-Statistical Methodology.

[ref-10] Chakrabarti M, Zhang N, Sauvage C, Munos S, Blanca J, Canizares J, Diez MJ, Schneider R, Mazourek M, McClead J, Causse M, Knaap Evander (2013). A cytochrome P450 regulates a domestication trait in cultivated tomato. Proceedings of the National Academy of Sciences of the United States of America.

[ref-11] Chen C, Zeng Z, Liu Z, Xia R (2018). Small RNAs, emerging regulators critical for the development of horticultural traits. Horticulture Research.

[ref-12] Chen XM (2004). A microRNA as a translational repressor of APETALA2 in Arabidopsis flower development. Science.

[ref-13] Cosgrove DJ (2015). Plant expansins: diversity and interactions with plant cell walls. Current Opinion in Plant Biology.

[ref-14] Cosgrove DJ (2018). Diffuse growth of plant cell walls. Plant Physiology.

[ref-15] Crawford BC, Nath U, Carpenter R, Coen ES (2004). CINCINNATA controls both cell differentiation and growth in petal lobes and leaves of Antirrhinum. Plant Physiology.

[ref-16] Czerednik A, Busscher M, Bielen BAM, Wolters-Arts M, De Maagd RA, Angenent GC (2012). Regulation of tomato fruit pericarp development by an interplay between CDKB and CDKA1 cell cycle genes. Journal of Experimental Botany.

[ref-17] Daccord N, Celton JM, Linsmith G, Becker C, Choisne N, Schijlen E, van de Geest H, Bianco L, Micheletti D, Velasco R, Di Pierro EA, Gouzy J, Rees DJG, Guerif P, Muranty H, Durel CE, Laurens F, Lespinasse Y, Gaillard S, Aubourg S, Quesneville H, Weigel D, van de Weg E, Troggio M, Bucher E (2017). High-quality de novo assembly of the apple genome and methylome dynamics of early fruit development. Nature Genetics.

[ref-18] Dahan Y, Rosenfeld R, Zadiranov V, Irihimovitch V (2010). A proposed conserved role for an avocado fw2.2-like gene as a negative regulator of fruit cell division. Planta.

[ref-19] Dare AP, Tomes S, Jones M, McGhie TK, Stevenson DE, Johnson RA, Greenwood DR, Hellens RP (2013). Phenotypic changes associated with RNA interference silencing of chalcone synthase in apple (Malus x domestica). The Plant Journal.

[ref-20] Dash M, Johnson LK, Malladi A (2012). Severe shading reduces early fruit growth in apple by decreasing cell production and expansion. Journal of the American Society for Horticultural Science.

[ref-21] Dash M, Johnson LK, Malladi A (2013). Reduction of fruit load affects early fruit growth in apple by enhancing carbohydrate availability, altering the expression of cell production-related genes, and increasing cell production. Journal of the American Society for Horticultural Science.

[ref-22] Dash M, Malladi A (2012). The AINTEGUMENTA genes, MdANT1 and MdANT2, are associated with the regulation of cell production during fruit growth in apple (Malus x domestica Borkh). BMC Plant Biology.

[ref-23] Dewitte W, Riou-Khamlichi C, Scofield S, Healy JM, Jacqmard A, Kilby NJ, Murray JA (2003). Altered cell cycle distribution, hyperplasia, and inhibited differentiation in Arabidopsis caused by the D-type cyclin CYCD3. The Plant Cell.

[ref-24] Di Marzo M, Herrera-Ubaldo H, Caporali E, Novak O, Strnad M, Balanza V, Ezquer I, Mendes MA, De Folter S, Colombo L (2020). SEEDSTICK controls arabidopsis fruit size by regulating cytokinin levels and FRUITFULL. Cell Reports.

[ref-25] Fornale S, Shi X, Chai C, Encina A, Irar S, Capellades M, Fuguet E, Torres JL, Rovira P, Puigdomenech P, Rigau J, Grotewold E, Gray J, Caparros-Ruiz D (2010). ZmMYB31 directly represses maize lignin genes and redirects the phenylpropanoid metabolic flux. The Plant Journal.

[ref-26] Franceschi PDe, Stegmeir T, Cabrera A, van der Knaap E, Rosyara UR, Sebolt AM, Dondini L, Dirlewanger E, Quero-Garcia J, Campoy JA, Iezzoni AF (2013). Cell number regulator genes in Prunus provide candidate genes for the control of fruit size in sweet and sour cherry. Molecular Breeding.

[ref-27] Frary A, Nesbitt TC, Frary A, Grandillo S, van der Knaap E, Cong B, Liu JP, Meller J, Elber R, Alpert KB, Tanksley SD (2000). fw2.2: a quantitative trait locus key to the evolution of tomato fruit size. Science.

[ref-28] Fraser PD, Enfissi EM, Halket JM, Truesdale MR, Yu D, Gerrish C, Bramley PM (2007). Manipulation of phytoene levels in tomato fruit: effects on isoprenoids, plastids, and intermediary metabolism. The Plant Cell.

[ref-29] Fukazawa J, Sakai T, Ishida S, Yamaguchi I, Kamiya Y, Takahashi Y (2000). REPRESSION OFSHOOT GROWTH, a bZIP transcriptional activator, regulates cell elongation by controlling the level of gibberellins. The Plant Cell.

[ref-30] Garcia-Hurtado N, Carrera E, Ruiz-Rivero O, Lopez-Gresa MP, Hedden P, Gong F, Garcia-Martinez JL (2012). The characterization of transgenic tomato overexpressing gibberellin 20-oxidase reveals induction of parthenocarpic fruit growth, higher yield, and alteration of the gibberellin biosynthetic pathway. Journal of Experimental Botany.

[ref-31] Gasser C (2015). miRNA pumps up the volume. Nature Plants.

[ref-32] Giovannoni JJ (2004). Genetic regulation of fruit development and ripening. The Plant Cell.

[ref-33] Guo HY, Wang YC, Wang LQ, Hu P, Wang YM, Jia YY, Zhang CR, Zhang Y, Zhang YM, Wang C, Yang CP (2017). Expression of the MYB transcription factor gene BplMYB46 affects abiotic stress tolerance and secondary cell wall deposition in Betula platyphylla. Plant Biotechnology Journal.

[ref-34] Guo M, Rupe MA, Dieter JA, Zou J, Spielbauer D, Duncan KE, Howard RJ, Hou Z, Simmons CR (2010). Cell number Regulator1 affects plant and organ size in maize: implications for crop yield enhancement and heterosis. The Plant Cell.

[ref-35] Guo M, Simmons CR (2011). Cell number counts–the fw2.2 and CNR genes and implications for controlling plant fruit and organ size. Plant Science.

[ref-36] Harada T, Kurahashi W, Yanai M, Wakasa Y, Satoh T (2005). Involvement of cell proliferation and cell enlargement in increasing the fruit size of Malus species. Scientia Horticulturae.

[ref-37] Hongo S, Sato K, Yokoyama R, Nishitani K (2012). Demethylesterification of the primary wall by PECTIN METHYLESTERASE35 provides mechanical support to the Arabidopsis stem. The Plant Cell.

[ref-38] Horiguchi G, Gonzalez N, Beemster GT, Inze D, Tsukaya H (2009). Impact of segmental chromosomal duplications on leaf size in the grandifolia-D mutants of Arabidopsis thaliana. The Plant Journal.

[ref-39] Hu Y, Xie Q, Chua NH (2003). The Arabidopsis auxin-inducible gene ARGOS controls lateral organ size. The Plant Cell.

[ref-40] Hu H, Zhang R, Tao Z, Li X, Li Y, Huang J, Li X, Han X, Feng S, Zhang G, Pen L (2018). Cellulose synthase mutants distinctively affect cell growth and cell wall integrity for plant biomass production in arabidopsis. Plant and Cell Physiology.

[ref-41] Inze D, De Veylder L (2006). Cell cycle regulation in plant development. Annual Review of Genetics.

[ref-42] Kim GT, Tsukaya H, Saito Y, Uchimiya H (1999). Changes in the shapes of leaves and flowers upon overexpression of cytochrome P450 in Arabidopsis. Proceedings of the National Academy of Sciences of the United States of America.

[ref-43] Krizek BA (2009). Making bigger plants: key regulators of final organ size. Current Opinion in Plant Biology.

[ref-44] Lavhale SG, Kalunke RM, Giri AP (2018). Structural, functional and evolutionary diversity of 4-coumarate-CoA ligase in plants. Planta.

[ref-45] Lee BH, Ko JH, Lee S, Lee Y, Pak JH, Kim JH (2009). The Arabidopsis GRF-INTERACTING FACTOR gene family performs an overlapping function in determining organ size as well as multiple developmental properties. Plant Physiology.

[ref-46] Libault M, Zhang X-C, Govindarajulu M, Qiu J, Ong YT, Brechenmacher L, Berg RH, Hurley-Sommer A, Taylor CG, Stacey G (2010). A member of the highly conserved FWL (tomato FW2.2-like) gene family is essential for soybean nodule organogenesis. Plant Journal.

[ref-47] Liscum E, Reed JW (2002). Genetics of Aux/IAA and ARF action in plant growth and development. Plant Molecular Biology.

[ref-48] Liu W, Zhang J, Jiao C, Yin X, Fei Z, Wu Q, Chen K (2019). Transcriptome analysis provides insights into the regulation of metabolic processes during postharvest cold storage of loquat (Eriobotrya japonica) fruit. Horticulture Research.

[ref-49] Livak KJ, Schmittgen TD (2001). Analysis of relative gene expression data using real-time quantitative PCR and the 2(T)(-Delta Delta C) method. Methods.

[ref-50] Ma C, Lu Y, Bai S, Zhang W, Duan X, Meng D, Wang Z, Wang A, Zhou Z, Li T (2014). Cloning and characterization of miRNAs and their targets, including a novel miRNA-Targeted NBS-LRR protein class gene in apple (Golden Delicious). Molecular Plant.

[ref-51] Malladi A (2020). Molecular physiology of fruit growth in apple. Horticultural Reviews.

[ref-52] Malladi A, Johnson LK (2011). Expression profiling of cell cycle genes reveals key facilitators of cell production during carpel development, fruit set, and fruit growth in apple (Malus ×domestica Borkh.). Journal of Experimental Botany.

[ref-53] Marin-Rodriguez MC, Orchard J, Seymour GB (2002). Pectate lyases, cell wall degradation and fruit softening. Journal of Experimental Botany.

[ref-54] Mehrtens F, Kranz H, Bednarek P, Weisshaar B (2005). The Arabidopsis transcription factor MYB12 is a flavonol-specific regulator of phenylpropanoid biosynthesis. Plant Physiology.

[ref-55] Menges M, Samland AK, Planchais S, Murray JA (2006). The D-type cyclin CYCD3;1 is limiting for the G1-to-S-phase transition in Arabidopsis. The Plant Cell.

[ref-56] Mizukami Y, Fischer RL (2000). Plant organ size control: AINTEGUMENTA regulates growth and cell numbers during organogenesis. Proceedings of the National Academy of Sciences of the United States of America.

[ref-57] Nibau C, Di Stilio VS, Wu HM, Cheung AY (2011). Arabidopsis and Tobacco superman regulate hormone signalling and mediate cell proliferation and differentiation. Journal of Experimental Botany.

[ref-58] Nole-Wilson S, Tranby TL, Krizek BA (2005). AINTEGUMENTA-like (AIL) genes are expressed in young tissues and may specify meristematic or division-competent states. Plant Molecular Biology.

[ref-59] Ogawa M, Hanada A, Yamauchi Y, Kuwalhara A, Kamiya Y, Yamaguchi S (2003). Gibberellin biosynthesis and response during Arabidopsis seed germination. The Plant Cell.

[ref-60] Pei MS, Cao SH, Wu L, Wang GM, Xie ZH, Gu C, Zhang SL (2020). Comparative transcriptome analyses of fruit development among pears, peaches, and strawberries provide new insights into single sigmoid patterns. BMC Plant Biology.

[ref-61] Pesquet E, Korolev AV, Calder G, Lloyd CW (2010). The microtubule-associated protein AtMAP70-5 regulates secondary wall patterning in arabidopsis wood cells. Current Biology.

[ref-62] Rhoades MW, Reinhart BJ, Lim LP, Burge CB, Bartel B, Bartel DP (2002). Prediction of plant microRNA targets. Cell.

[ref-63] Ripoll JJ, Bailey LJ, Mai Q-A, Wu SL, Hon CT, Chapman EJ, Ditta GS, Estelle M, Yanofsky MF (2015). microRNA regulation of fruit growth. Nature Plants.

[ref-64] Roach MJ, Mokshina NY, Badhan A, Snegireva AV, Hobson N, Deyholos MK, Gorshkova TA (2011). Development of cellulosic secondary walls in flax fibers requires beta-galactosidase. Plant Physiology.

[ref-65] Schaller GE, Street IH, Kieber JJ (2014). Cytokinin and the cell cycle. Current Opinion in Plant Biology.

[ref-66] Scheible WR, Pauly M (2004). Glycosyltransferases and cell wall biosynthesis: novel players and insights. Current Opinion in Plant Biology.

[ref-67] Schiessl K, Kausika S, Southam P, Bush M, Sablowski R (2012). JAGGED controls growth anisotropy and coordination between cell size and cell cycle during plant organogenesis. Current Biology.

[ref-68] Schruff MC, Spielman M, Tiwari S, Adams S, Fenby N, Scott RJ (2006). The AUXIN RESPONSE FACTOR 2 gene of Arabidopsis links auxin signalling, cell division, and the size of seeds and other organs. Development.

[ref-69] Shannon P, Markiel A, Ozier O, Baliga NS, Wang JT, Ramage D, Amin N, Schwikowski B, Ideker T (2003). Cytoscape: a software environment for integrated models of biomolecular interaction networks. Genome Research.

[ref-70] Shinozaki Y, Nicolas P, Fernandez-Pozo N, Ma Q, Evanich DJ, Shi Y, Xu Y, Zheng Y, Snyder SI, Martin LBB, Ruiz-May E, Thannhauser TW, Chen K, Domozych DS, Catala C, Fei Z, Mueller LA, Giovannoni JJ, Rose JKC (2018). High-resolution spatiotemporal transcriptome mapping of tomato fruit development and ripening. Nature Communications.

[ref-71] Sugimoto-Shirasu K, Roberts K (2003). Big it up: endoreduplication and cell-size control in plants. Current Opinion in Plant Biology.

[ref-72] Trapnell C, Pachter L, Salzberg SL (2009). TopHat: discovering splice junctions with RNA-Seq. Bioinformatics.

[ref-73] Vanneste S, Friml J (2009). Auxin: a trigger for change in plant development. Cell.

[ref-74] Wang L, Tang W, Hu Y, Zhang Y, Sun J, Guo X, Lu H, Yang Y, Fang C, Niu X, Yue J, Fei Z, Liu Y (2019). A MYB/bHLH complex regulates tissue-specific anthocyanin biosynthesis in the inner pericarp of red-centered kiwifruit Actinidia chinensis cv. Hongyang. The Plant Journal.

[ref-75] Wang Y-c, Wang N, Xu H-f, Jiang S-h, Fang H-c, Su M-y, Zhang Z-y, Zhang T-l, Chen X-s (2018). Auxin regulates anthocyanin biosynthesis through the Aux/IAA-ARF signaling pathway in apple. Horticulture Research.

[ref-76] Whiting MD, Ophardt D, McFerson JR (2006). Chemical blossom thinners vary in their effect on sweet cherry fruit set, yield, fruit quality, and crop value. HortTechnology.

[ref-77] Wolf S, Mouille G, Pelloux J (2009). Homogalacturonan methyl-esterification and plant development. Molecular Plant.

[ref-78] Xu C, Shen Y, He F, Fu X, Yu H, Lu W, Li Y, Li C, Fan D, Wang HC, Luo K (2019). Auxin-mediated Aux/IAA-ARF-HB signaling cascade regulates secondary xylem development in Populus. New Phytologist.

[ref-79] Yao J-L, Tomes S, Xu J, Gleave AP (2016). How microRNA172 affects fruit growth in different species is dependent on fruit type. Plant Signaling & Behavior.

[ref-80] Yao J-L, Xu J, Cornille A, Tomes S, Karunairetnam S, Luo Z, Bassett H, Whitworth C, Rees-George J, Ranatunga C, Snirc A, Crowhurst R, De Silva N, Warren B, Deng C, Kumar S, Chagne D, Bus VGM, Volz RK, Rikkerink EHA, Gardiner SE, Giraud T, MacDiarmid R, Gleave AP (2015). A microRNA allele that emerged prior to apple domestication may underlie fruit size evolution. Plant Journal.

[ref-81] Yi K, Menand B, Bell E, Dolan L (2010). A basic helix-loop-helix transcription factor controls cell growth and size in root hairs. Nature Genetics.

[ref-82] Yu S, Galvao VC, Zhang YC, Horrer D, Zhang TQ, Hao YH, Feng YQ, Wang S, Schmid M, Wang JW (2012). Gibberellin regulates the Arabidopsis floral transition through miR156-targeted SQUAMOSA promoter binding-like transcription factors. The Plant Cell.

[ref-83] Zeng JK, Li X, Xu Q, Chen JY, Yin XR, Ferguson IB, Chen KS (2015). EjAP2-1, an AP2/ERF gene, is a novel regulator of fruit lignification induced by chilling injury, via interaction with EjMYB transcription factors. Plant Biotechnology Journal.

[ref-84] Zhou Z, Cong P, Tian Y, Zhu Y (2017). Using RNA-seq data to select reference genes for normalizing gene expression in apple roots. PLOS ONE.

[ref-85] Zhou Z, Tian Y, Cong P, Zhu Y (2018). Functional characterization of an apple (& IT;Malus & IT;x & IT; domestica & IT;) LysM domain receptor encoding gene for its role in defense response. Plant Science.

[ref-86] Zhou Z, Zhu Y, Tian Y, Yao JL, Bian S, Zhang H, Zhang R, Gao Q, Yan Z (2021). MdPR4, a pathogenesis-related protein in apple, is involved in chitin recognition and resistance response to apple replant disease pathogens. Journal of Plant Physiology/TD.

[ref-87] Zhu QH, Helliwell CA (2011). Regulation of flowering time and floral patterning by miR172. Journal of Experimental Botany.

