# Supplementary material for: Transcriptome analysis of transgenic apple fruit overexpressing microRNA172 reveals candidate transcription factors regulating apple fruit development at early stages
Source: PeerJ. 2021 Dec 22;9:e12675. doi: 10.7717/peerj.12675 (PMC8710058; doi:10.7717/peerj.12675)
Supplement: Supplemental Information 3 [file peerj-09-12675-s003.docx]

**Supplementary figures**

**Figure S1**. **Pearson correlation between samples.**

**
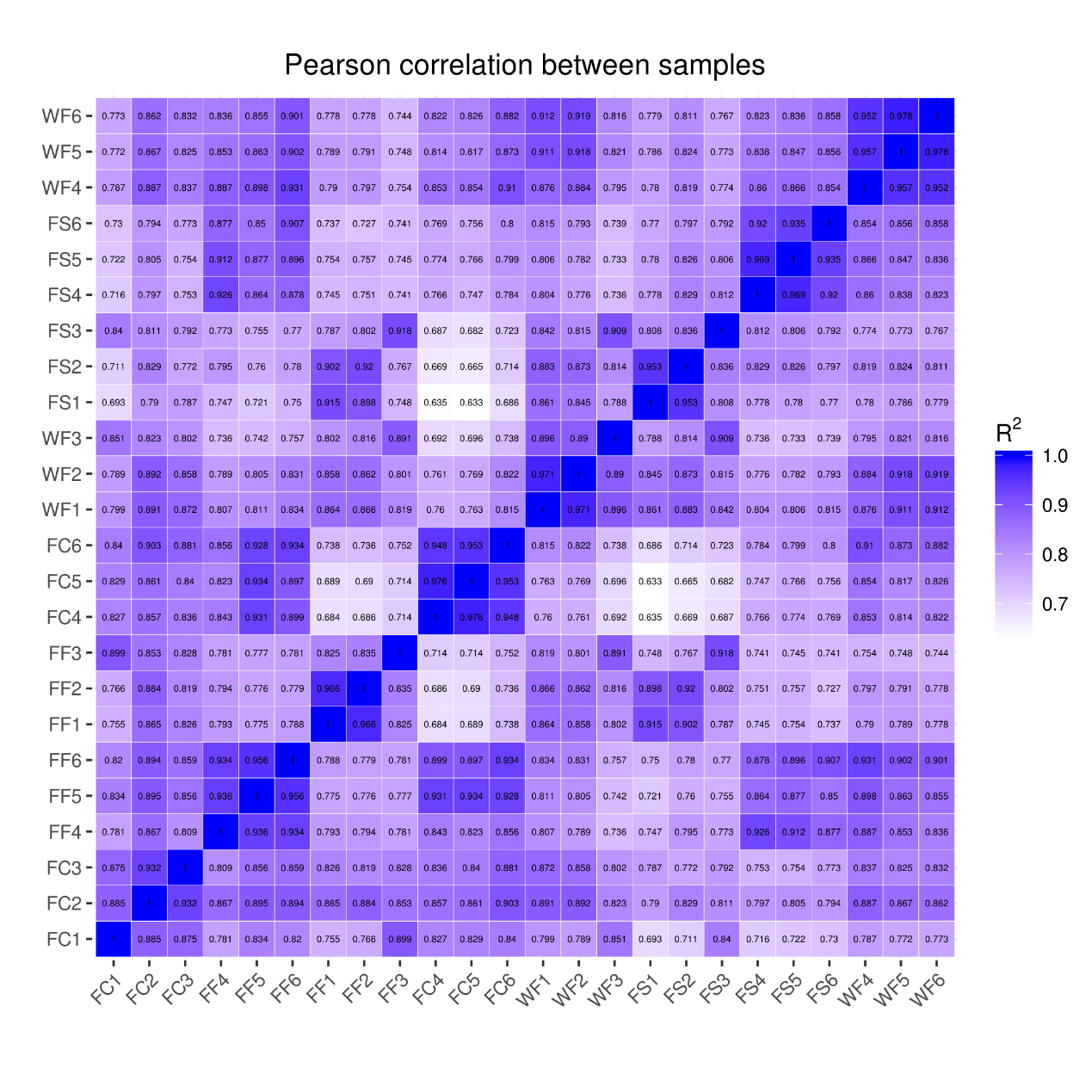
Figure S2. Phylogenetic tree of 4CL proteins of apple and other species.**

**
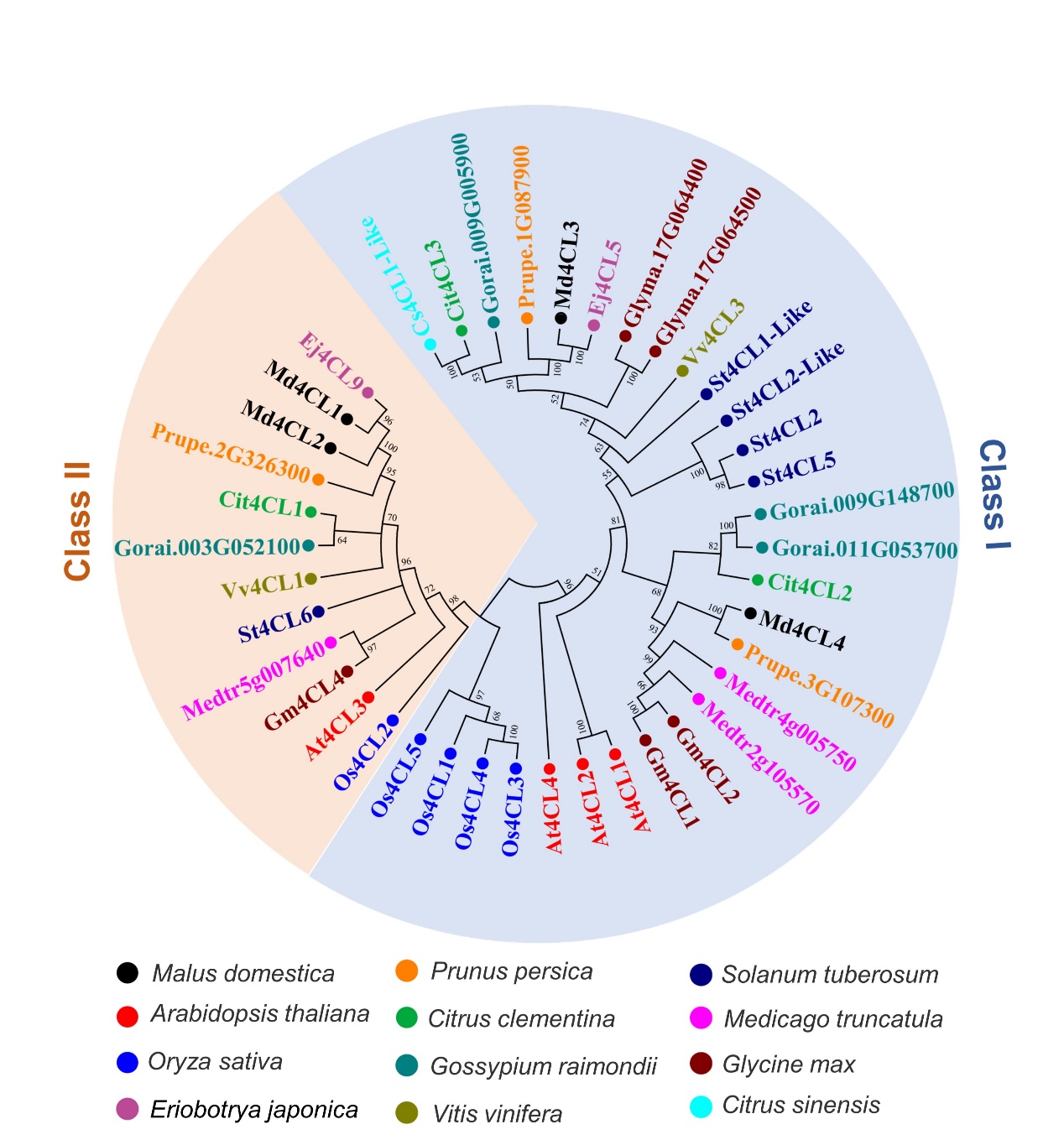
**
